# A Case of Traumatic Emphysema

**Published:** 1897-12

**Authors:** 


A Case of Traumatic Emphysema.—On October 18, 1896, I was summoned by a farmer, who informed me that one of his cows was so inflated that he, for fear that she might suffocate, had punctured the right side with a trocar found in the neighborhood. As absolutely no air escaped, he punctured the left side. A great quantity of air escaped, but the cow still looked as if she had been "blown up." I arrived about three hours after this operation had been performed, and the enormous size of the animal was very striking. The expresssion, "blown up," exactly characterized her appearance. On the right and left side of the abdominal cavity the round edges of a wound, matted with bloody hair, were plainly visible. On the right side beneath the point of puncture a strip of skin as broad as the hand had been raised up (inflated). This strip ran up over the back from just beneath the shoulder-blade and along the left side from the outer corner of the haunch-bone across the belly, breast, back, and shoulder as far as the fifth vertebra of the neck, then down again on the lateral side as far as the carpal-joint. On feeling it with the hand crackling could be plainly heard, and the frequent bellowing showed that the animal was in pain. Impressions with the finger soon disappeared. The surface of the skin was moderately warm. The temperature taken at the rectum was 39.5° C. The pulse was strong at 56 to the minute. On auscultation the single heart-beats could be distinctly heard, but there was no distinct sound audible in respiration, which was about 45 per minute, accompanied by a strong expansion of the chest. Digestive activity seemed to have ceased entirely. Both forelegs were spread out sidewise from the chest, and all efforts to cause the animal to change its position were fruitless.

To allay the emphysema I had linen cloths wrung out in cold water and laid upon the puffed-up tract every three or four hours. Over these a blanket was fastened with a girdle. Later, clysters of soapy water were applied with the syringe.

The next day the emphysema extended only on the right side across the back, and on the left across the breast and shoulders, including the elbows. The temperature, however, had risen to 40.8° C., crackling could be heard when the affected parts were touched. The respiration was 36, the pulse 52 to the minute. Food, consisting of liquid meal and bran, was entirely, though slowly, consumed.

In the following days the emphysema decreased slowly and steadily, and the general condition showed no signs of disturbance after the 20th. By the 28th the emphysema had entirely disappeared.—Speer, in *Deutsche Thierarztliche Wochenschrift*, Jan. 30, 1897.

